# Enhancing the Ignition, Hardness and Compressive Response of Magnesium by Reinforcing with Hollow Glass Microballoons

**DOI:** 10.3390/ma10090997

**Published:** 2017-08-25

**Authors:** Vyasaraj Manakari, Gururaj Parande, Mrityunjay Doddamani, Manoj Gupta

**Affiliations:** 1 Department of Mechanical Engineering, National University of Singapore, 9 Engineering Drive 1, Singapore 117576, Singapore; mbvyasaraj@u.nus.edu (V.M.); gururaj.parande@u.nus.edu (G.P.); 2 Advanced Manufacturing Laboratory, Department of Mechanical Engineering, National Institute of Technology Karnataka, Surathkal 575025, India; mrdoddamani@nitk.edu.in

**Keywords:** magnesium, syntactic foam, glass microsphere, compression, ignition, microstructure

## Abstract

Magnesium (Mg)/glass microballoons (GMB) metal matrix syntactic foams (1.47–1.67 g/cc) were synthesized using a disintegrated melt deposition (DMD) processing route. Such syntactic foams are of great interest to the scientific community as potential candidate materials for the ever-changing demands in automotive, aerospace, and marine sectors. The synthesized composites were evaluated for their microstructural, thermal, and compressive properties. Results showed that microhardness and the dimensional stability of pure Mg increased with increasing GMB content. The ignition response of these foams was enhanced by ~22 °C with a 25 wt % GMB addition to the Mg matrix. The authors of this work propose a new parameter, ignition factor, to quantify the superior ignition performance that the developed Mg foams exhibit. The room temperature compressive strengths of pure Mg increased with the addition of GMB particles, with Mg-25 wt % GMB exhibiting the maximum compressive yield strength (CYS) of 161 MPa and an ultimate compressive strength (UCS) of 232 MPa for a GMB addition of 5 wt % in Mg. A maximum failure strain of 37.7% was realized in Mg-25 wt % GMB foam. The addition of GMB particles significantly enhanced the energy absorption by ~200% prior to compressive failure for highest filler loading, as compared to pure Mg. Finally, microstructural changes in Mg owing to the presence of hollow GMB particles were elaborately discussed.

## 1. Introduction

Magnesium (Mg) contributes ~2.7% by weight to the earth’s crust, making it the sixth most abundant element [1]. Mg-based materials show enormous potential in weight-sensitive applications, as they are the lightest structural material available. Mg has a density of only 1.74 g/cc which is ~33%, ~61% and ~77% lower than that of aluminium (Al), titanium (Ti), and iron (Fe), respectively [2]. Increased demand for lightweighting drives the interest in Mg-based materials to be used in a variety of structural components in sectors striving for weight reduction, higher fuel efficiency, and payload capacity, such as automotive, aerospace, and defense. In addition to its light weight, Mg-based materials also exhibit better specific mechanical properties, damping characteristics, and impact resistance [3]. However, Mg exhibits low corrosion resistance, room temperature ductility, and high temperature strength, which limits its wide scale adaptability [4,5]. These facts necessitate increasing thrust in developing Mg-based syntactic foams with superior specific mechanical properties.

Metal matrix syntactic foams (MMSFs) are produced by dispersing hollow particulate fillers in the metal matrix. Incorporating porosity through hollow particles instead of embedding air/gas voids provides a reinforcing effect to each pore, and imparts properties similar or superior to what would be found in monolithic cellular materials, in particular metallic foams [6]. The incorporation of porosity in a material makes the material lightweight and enhances the compressibility. Energy absorption and ductility can be further enhanced by increasing the extent of porosity. Open cell foams lack in strength and modulus, which limits their application. MMSFs exhibit superior mechanical properties for the same amount of porosity due to their closed cell structure [7]. Further, syntactic foams offer an interesting and wide spectrum of properties, such as lower densities, low moisture uptake, high specific stiffness, superior blast and energy absorption, low thermal conductivity, and better dimensional stability, which would make them ideal for many applications in ground vehicle transportation, aerospace, defense, electronic packaging, biomedical, and marine applications [8]. Recent review articles have detailed the synthesis methods, microstructures, mechanical properties and applications of Mg, Al, and Ti matrix syntactic foams [7,8]. Engineered hollow spheres of SiC and Al_2_O_3_ [9,10], inexpensive fly ash cenospheres, and hollow glass microballoons (GMB) [11,12,13,14,15] have been used as fillers in developing these syntactic foams.

Though Mg is expanding into more promising lightweight regime and medical applications, very little attention has been given to studies on Mg matrix syntactic foams (MgMSFs) compared to Al and Ti syntactic foams. This is likely due to the high reactivity of Mg and processing challenges that can lead to the microballoon breakage and related infiltration issues. From the open literature, a limited number of Mg alloy systems (AZ31B, AM 20, AM 50, AZ91, AZ91D and ZC63) were used for the synthesis of MgMSFs [7,8], and only few publications on monolithic Mg-based syntactic foams are available [16,17]. Fly ash cenospheres are widely used as fillers in these studies to synthesize syntactic foams because of their low cost. Cenospheres are recovered from coal combustion ash and are available in large quantities in thermal power plants. However, being waste by-products, they contain numerous defects in their structures, which is also reflected in the properties of developed foams. As a result, studies have experimented with the engineered hollow particles of ceramics such as SiC and Al_2_O_3_ for high-performance applications. Recently, a Mg-AZ91 matrix reinforced with SiC particles has been developed by Anantharaman et al. [9] and Rivero et al. [18] with a density as low as 0.92 g/cc and were found to have better performance than Al matrix syntactic foams (AlMSFs) on unit weight basis, wherein MgMSFs showed 44% higher specific compressive strength as compared to AlMSFs.

Syntactic foams made of Mg and hollow GMB have not been explored so far. In recent studies, the compressive and corrosion properties of AZ91D/sodalime–borosilicate GMB syntactic foams [19] were characterized. The compressive properties of the AZ91D matrix showed an increase in compressive yield strength (CYS) from 112 to 143, 161 and 168 MPa with 15, 20 and 23 wt %, GMB additions respectively. A similar trend was observed in ultimate compressive strength (UCS) with a maximum of 243 MPa (~52% rise). Also, the addition of GMB particles caused a decrease in the α-Mg phase, which resulted in improved corrosion resistance for the syntactic foams. Nevertheless, this study dealt increasing the processing complexities of an Mg alloy and quantifying additional phase formations. Also, it has been demonstrated that the sodalime-borosilicate hollow glass microspheres (GMB) used in most applications are susceptible to significant degradation under high temperature and long-term exposures [20,21]. In response to concerns over the long-term stability of sodalime-borosilicate filled syntactic foams, GMB comprised of borosilicate glass or silica can be used in developing MgMSFs. Particles with this glass chemistry have been demonstrated to be inert to degradation in wet environments and also presents a number of other potential benefits for use in syntactic foams, as the glass chemistry has lower thermal expansion and a higher softening temperature as compared with sodalime-borosilicate glass [20]. The present work is aimed at exploring the possibility of using silica GMB for the development of high performance Mg-based syntactic foams using a disintegrated melt deposition (DMD) approach.

For fabricating MgMSFs, pressure infiltration, stir casting, and conventional powder metallurgy techniques are currently used as the main processing techniques [8]. Each technique has its own advantages and limitations for the development of MgMSFs. The limitations of infiltration technique include the fracturing of hollow particles, matrix metal filling inside the spheres due to high infiltration pressure, and high residual porosity due to low infiltration pressure. Wettability and undesired phase formation between the matrix/sphere interface and the permeability of the reinforcement bed are also challenging issues. The major drawback of the stir-casting technique is flotation of low-density hollow particles and particle fracture. This technique is also particularly sensitive to segregation and agglomeration of the hollow reinforcement. The major issue associated with the powder metallurgy technique is the breakage of hollow particles at high volume fractions of the reinforcement. Therefore, to successfully develop MgMSFs, the use of different or new processing techniques and/or optimization of processing parameters, theoretically and experimentally, are still needed. DMD is a unique cost-effective technique that brings together the advantages of conventional casting and spray processing, utilizing lower impinging gas jet velocities, and higher superheat temperatures to produce bulk composite material. The advantage of DMD is the ability to obtain a uniform distribution of secondary reinforcements and equiaxed grains, resulting in a highly dense material with enhanced properties [22].

Reinforcing pure Mg with glass microballoons using a DMD route of processing is quite a challenging and interesting task, hence adopted in this work. Mg/GMB foams are investigated for coefficient of thermal expansion (CTE), hardness, ignition, elastic modulus, and compressive properties. The GMB amount is varied in the Mg matrix by 5, 15 and 25 wt % which corresponds to ~8, 22.6 and 35.5 vol %. Pure Mg samples are also casted for comparison. Structure–property correlations are elaborately discussed with micrography.

## 2. Materials and Methods

### 2.1. Materials

Mg in turnings form (ACROS Organics, Morris Plains, NJ, USA) with 99.9% purity were used as matrix material. Hollow GMB particles with a mean particle diameter of 11 µm and a density of ~1.05 g/cc procured from Sigma Aldrich, Singapore, are used as reinforcing filler.

### 2.2. Processing

DMD technique was used to synthesize Mg/GMB syntactic foams. This method involves adding the raw material (i.e., Mg and GMB particles) in alternate layers to form a sandwich pattern in a graphite crucible, and heating it in an electrical resistance furnace to 720 °C in a protective inert argon gas atmosphere. This method employs a combination of vortex stirring of melt at 465 rpm for 5 min, followed by the subsequent disintegration by two jets of argon (Ar) gas to obtain a good yield, with a uniform dispersion of hollow GMB in the Mg matrix [23]. The stirrer used is a mild steel impeller with twin blade (pitch 45°) coated with Zirtex 25 (86% ZrO_2_, 8.8% Y_2_O_3_, 3.6% SiO_2_, 1.2% K_2_O and Na_2_O, with 0.3% trace inorganic) in order to avoid contamination of molten metal with iron. The melt, released through an orifice of 10 mm diameter located at the crucible’s base, was disintegrated by two argon gas jets at a flow rate of 25 litres per minute (lpm) that were oriented normal to the melt stream. The disintegrated melt is then deposited on the substrate, forming an ingot of 40-mm diameter, which was subsequently trimmed to required dimensions for experimental investigations. All of the samples were coded with Mg-XX convention, where XX represents the weight % of GMB.

### 2.3. Methods

Density measurements were made in accordance with the Archimedes’ principle on five samples. Distilled water was used as the immersion fluid. The samples were weighed using an A&D ER-182A electronic balance (Bradford, MA, USA) having an accuracy of ±0.0001 g.

Coefficients of thermal expansion (CTE) of the pure Mg and foam samples were determined by measuring the displacement of the samples as a function of temperature in the temperature range of 50–350 °C using an automated TMA PT1000 thermo-mechanical analyzer (Tokyo, Japan).

The ignition temperature of pure Mg and foam samples (2 × 2 × 1 mm^3^) were analyzed using a Shimadzu DTG-60H Thermo Gravimetric Analyzer (Kyoto, Japan). The samples were heated from 30 °C to 750 °C at a heating rate of 10 °C/min in purified air with a flow rate of 50 mL/min. The point at which a rapid increase in the weight of the sample is triggered (as a result of sharp oxidation upon ignition) is considered the ignition temperature [1]. After the sample was burnt out, the temperature rate was restored to the initial set-value. The crucible was removed immediately after the test, cooled sufficiently in order to avoid the contamination of TGA and overflow of the ignited powder from the sample. Four replicates were tested of each composition to ensure repeatability, and the average values are reported.

Microstructural characterization studies were conducted on metallographically polished samples to investigate morphological characteristics and the presence of second phases, if any. A Field Emission Scanning Electron Microscope (Hitachi FESEM-S4300, Hitachi Ltd., Tokyo, Japan) equipped with an energy dispersive spectrometer (EDS) was used to perform micrography.

Microhardness measurements were carried out on flat and polished specimens using a Shimadzu HMV automatic digital microhardness tester (Kyoto, Japan) that had a Vickers indenter with a phase angle of 136°. The samples were subjected to a load of 25 gf for a dwell time of 15 s. Test was conducted as per ASTM E384-16 [24].

The room temperature compressive properties of pure Mg and foam samples were determined as per ASTM E9-09 [25] using an MTS-810 testing machine (Eden Prairie, MN, USA) with a strain rate of 10^−4^ s^−1^. Test specimens of Φ 8 × 8 mm were used. For each composition, five specimens were tested to ensure the repeatability of the results.

The elastic modulus of Mg and their foams with dimensions of Φ 7 × 60 are estimated using the resonant frequency and damping analyser (IMCE, Genk, Belgium), as outlined in ASTM E1876-15 [26]. The sample was excited by a light impact to bending vibrations of the first mode at low amplitudes. Two polymer wires placed in the nodes of the vibration mode support the sample to avoid background damping. The specimen vibrations were recorded by a microphone. The software detects the resonance frequency for calculating the elastic modulus of the specimens.

## 3. Results and Discussion

### 3.1. Microstructural Characterization

**Figure 1:**
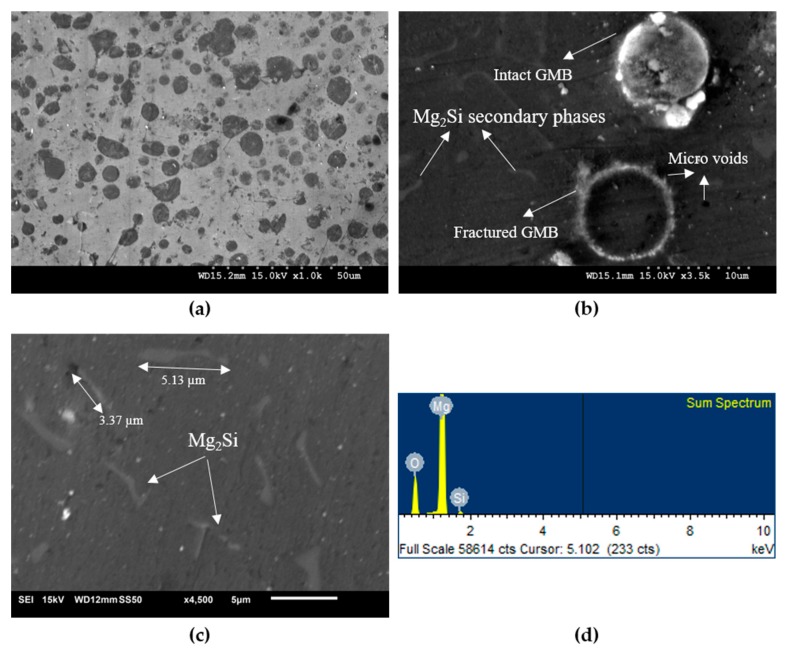
(**a**) Representative microstructure of Mg-15 foam; (**b**) GMB showing uniform wall thickness (**c**) Mg_2_Si dendrites present in the Mg matrix and (**d**) Energy dispersive spectrometer (EDS) result indicating the presence of Mg_2_Si as secondary phases.

Micrography was carried out on cast samples of Mg syntactic foams, and is presented in Figure 1. Micrography revealed a uniform distribution of intact GMB particles, secondary phase particles, and the presence of microvoids at the particles/Mg matrix interface. GMB/secondary particles are fairly well distributed, and the magnitude of secondary phase formation is observed to increase with higher filler loadings. The hollow glass microspheres are noted to be having an outer diameter of 8–13 µm, and exhibited good morphology variations within the microstructure (Figure 1a). Limited reaction zones are also clearly evident along the walls of the glass spheres, which could potentially lead to strong interfacial bonding between the foam constituents. Minimally fractured GMB particles due to interface reactions between the Mg matrix and GMB and processing parameters were also observed (Figure 1b).

A careful investigation of the chemical reaction products during the DMD process was also carried out by analyzing the presence of surface chemical elements using EDS (Figure 1d). Mg has a high reaction activity; therefore, when GMB particles are added to the Mg melt, the possibility of a chemical reaction between Mg elements and the silica phase present in GMB is inevitable. Thermodynamic computation indicates that the following chemical reaction is likely to happen:

2Mg + SiO_2_ → Si + 2MgO (1)

2Mg + Si → Mg_2_Si (2)

EDS revealed the presence of Mg_2_Si as a secondary phase in Mg/GMB foam (Figure 1d). Mg_2_Si formed, and can grow from the particle shell into the matrix. The diffusion of a Si atom to the Mg matrix might be the cause for the formation of Mg_2_Si having two different morphologies, namely, dendrite crystals and polygons. Mg_2_Si dendrites formed are observed to be in the size range of 3–5 µm.

Very few GMB are fractured, owing to the processing route followed as seen from the micrograph presented in Figure 1a. The Mg matrix occupies the space created due to microballoon fracture, and may compromise the overall density of the foam.

### 3.2. Density Measurement

The values of experimental densities based on the Archimedes Principle are presented in Table 1. The results show that the addition of GMB particles led to a significant reduction in density (13.4%). Foam density decreases with increasing GMB content. Also, it can be noticed that the experimental density values are less than the theoretical ones, indicating that the GMB particles are mostly intact in the Mg matrix, signifying the suitability of the DMD processing route in developing MgMSFs. It is also observed that the Mg/GMB foams synthesized in this study exhibit the densities closer to polymer-based composites such as those in PE-30% fiber glass composite (1.429 g/cc) [27]. These facts make Mg foams suitable for use in wider applications operating in high-temperature environments.

### 3.3. Coefficient of Thermal Expansion (CTE)

The coefficient of the thermal expansion values for pure Mg and Mg/GMB syntactic foams are listed in Table 1. The CTE of the syntactic foams decreases with the increasing GMB loading, providing improved dimensional stability to the developed foams. The CTE values for Mg-5 GMB (24.2 × 10^−6^/K), Mg-15 (22.7 × 10^−6^/K) and Mg-25 (21.2 × 10^−6^/K) were found to be ~10.7%, ~16.2%, and ~21.7% lower than that of pure Mg (27.1 × 10^−6^/K). This linear decreasing trend in the CTE values can be attributed to the lower CTE value of hollow GMB (4–8 × 10^−6^/K) [20,28], and reasonably good interfacial integrity between the Mg matrix and GMB (Figure 1a). The results of CTE measurement are found to be in accordance with the theory that the thermal expansion of composites is governed by the competing interactions of Mg matrix expansion and the constraint of reinforcement particles through their interfaces.

Mg/GMB syntactic foams having combination of low density and CTE can make them potential materials for degradable fracture ball applications in oil and gas reservoirs, where polymers such as Poly(ether ether ketone) (PEEK) and Torlon^®^ are currently being used [29]. Also, a low CTE along with dimensional stability at high temperatures make Mg syntactic foams viable for electronic packaging applications, where tailoring CTE over a wide range can be useful. Further, improvements in the dimensional stability of Mg syntactic foams at reduced density levels can lead to improved performance in aircraft components upon encountering low temperatures at high altitudes and frictional heating during rapid descent.

### 3.4. Ignition Properties

The onset of ignition occurs only when the stable surface oxide of Mg-based materials tends to lose its protective properties [30]. Pure Mg can auto-ignite in solid state due to the rapid increase in localized heat, which causes melting and evaporation of the metal locally. When the Mg vapor is in contact with air at the gas/metal interface, the metal ignites. However, with the modification of the chemistry of the material by the addition of thermally stable alloying elements and reinforcements, the mechanism changes and can delay the onset of ignition [31]. GMB are already being employed in thermoplastics as a filler to achieve flame retardancy capabilities and thermal stability [32,33]. Therefore, an attempt has been made to study and analyse the effect of thermally stable GMB on the ignition temperature of monolithic Mg. The present work is the first attempt towards exploring the potential of MgMSFs for ignition resistant applications.

The ignition results using TGA (thermogravimetric analysis) are presented in Table 1. The ignition temperature of pure Mg increases progressively with the addition of GMB, with Mg-25 syntactic foam exhibiting the maximum ignition temperature of 612 °C, which is ~22 °C higher than that of Mg (590 °C). The ignition temperature for Mg-25 foam (612 °C) is found to be higher than most of the commercially available Mg alloys, such as AZ61 (559 °C), AZ63 (573 °C), AZ91(600 °C), AM50 (585 °C), AM60 (525 °C), ZK40A (500 °C), ZK51A (552 °C), and ZK60A (499 °C) [31]. The ascending behavior of the point of ignition, and the enhanced resistance to ignition with an increasing volume fraction of GMB may be attributed to the lower CTE values, thus maintaining the thermal and dimensional stability of the Mg foams. In addition, GMB with Si-O structure have a large surface area and lower density tend to accumulate near the regressing sample surface without sinking. This can protect the inner matrix by reducing the specific areas of oxidation and hence delaying the onset of ignition in Mg/GMB syntactic foams [32,33]. Further, the Pilling–Bedworth ratio for SiO_2_ is 1.89, which indicates that a protective layer can be formed on the surface of Mg, restraining its reaction with oxygen [34].

In a recent article studying the ignition response of Mg-SiO_2_ nanocomposites [35], it was observed that the ignition temperature increased linearly with the decrease in the thermal conductivity of the material. The presence of nanoreinforcements in the Mg matrix acted as insulating sites, thereby delaying the onset of ignition in pure Mg. Further, the thermal conductivity of the composites, k (W/m. K), is directly related to the amount of reinforcement added to the matrix. In view of the ability of reinforcement to reduce the availability of metallic matrix for ignition and the thermal conductivity of the material, the authors propose a new parameter referred to as ignition factor (IF). IF can be defined as the ratio of thermal conductivity of the composite to the thermal conductivity of the matrix on the overall available volume of the matrix material, Vm (by reducing the volume percent of reinforcement), and is given by:

(3) $$\mathrm{Ignition}\;\mathrm{Factor}\;\left(\mathrm{IF}\right)=V_{m}\times (\frac{k_{\mathrm{composite}}}{k_{\mathrm{matrix}}})$$

Thermal conductivity, k (W/m·K), at 400 °C for the composites was calculated theoretically by the rule of mixtures using theoretical values of Mg (135 (W/m·K)) and GMB (0.2 (W/m·K)), which is expressed as:

α_c_ = α_m_. v_m_ + α_p_. v_p_ (4)

where α is the thermal conductivity, k (W/m·K); v is the volume fraction; and subscripts c, m, and p refer to the composite, matrix, and reinforcement phase, respectively. The thermal conductivity values for Mg-5, Mg-15, and Mg-25 GMB are calculated to be 124.22, 104.535, and 87.146 W/m·K, respectively.

**Figure 2:**
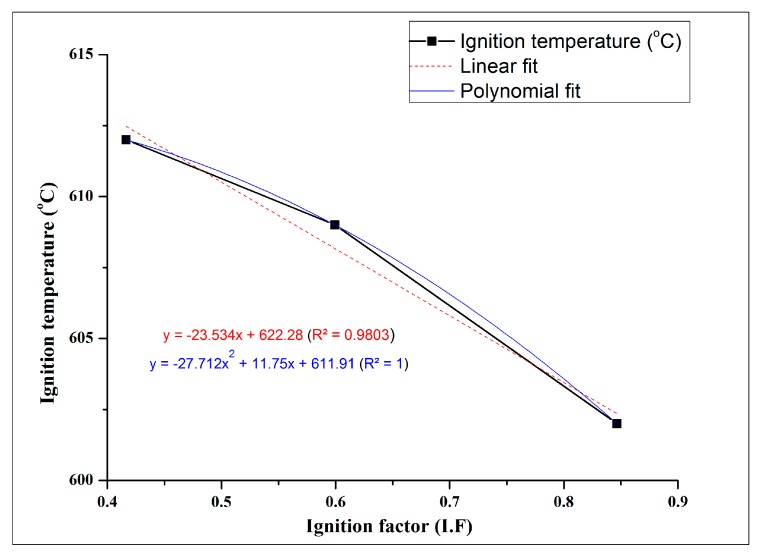
Ignition temperature versus ignition factor (IF) of the synthesized Mg/GMB syntactic foams.

The computed IF is correlated with the ignition temperature for the Mg/GMB foams to understand the effect of increased insulating sites on the overall ignition enhancement of the syntactic foams. The ignition temperature shows a perfect first order polynomial fit with IF, as seen from Figure 2. This can be expressed using Equation (4) as:

T_i_ = −27.712 (IF)^2^ + 11.75 IF + 611.91 (R^2^ = 1) (5)

This suggests that is not only the thermal conductivities that play a major role in the ignition behaviour of the syntactic foams, but also the relative volume of the matrix. GMB being dispersed uniformly in the Mg matrix act as insulating sites that exhibit superior ignition performance.

With the ban on the use of Mg-based materials being lifted by the FAA in 2015 in aerospace applications, there is a renewed interest in replacing Al alloy-based materials in the aircraft components. Mg/GMB syntactic foams with enhanced specific properties and ignition resistance can be potential materials in such applications. However, the underlying dominating mechanisms determining the ignition temperatures of Mg-based materials are complex and not very well known, particularly in syntactic foams. Therefore, further study on the mechanisms that affect the increase/decrease in the ignition temperature with the presence of GMB, and the dependence of the ignition characteristics on the volume fraction and wall thickness of GMB, will be an interesting direction of future research.

### 3.5. Microhardness

The microhardness measurements for pure Mg and their syntactic foams are presented in Table 2. The progressive addition of GMB particles resulted in a steady rise in the hardness of pure Mg. With the addition of 25 wt % GMB, a maximum average value of 107 Hv was noted, which is ~127.7% higher than that of pure Mg (47 Hv). This is in agreement with the published literature, where Anbuchezhiyan et al. [19] observed an increase in hardness with the addition of GMB to a AZ91D matrix. GMB particles restrain themselves from deformation in addition to constraining Mg’s plastic deformation, resulting into such a behaviour.

### 3.6. Compression Properties and Fracture Behaviour

**Figure 3:**
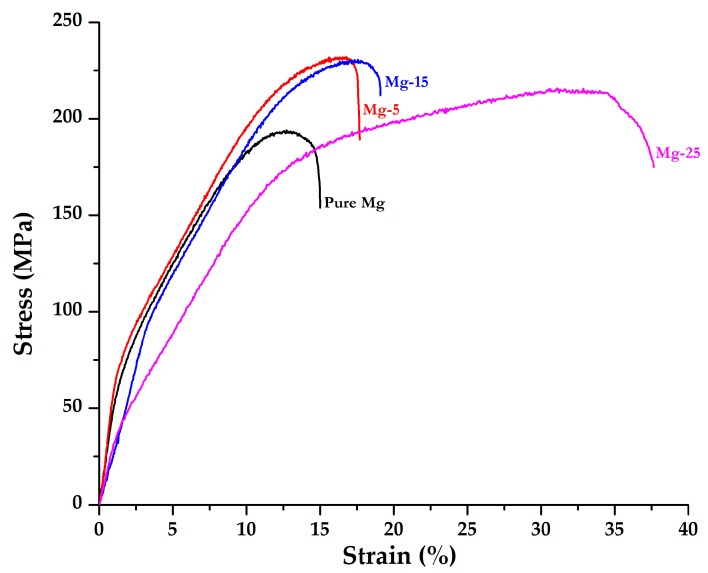
Engineering stress-strain curves of pure Mg and the synthesized Mg/GMB syntactic foams during compressive loading.

Representative stress-strain curves from quasi-static compression testing of Mg/GMB syntactic foams are presented in Figure 3. The stress-strain behaviour of the Mg-25 samples is similar to those commonly observed for metal and polymer syntactic foams [18,36]. The curve has an initial elastic region, followed by a post-yield plastic deformation at a relatively constant stress level (plateau region). Compressive behaviour did not show any distinct yield point. The onset of a plateau region post-yielding indicates no significant collapse in the matrix structure or after GMB failure. On the contrary, Mg, Mg-5, and Mg-15 do not exhibit the similar behavior. The upward concave (sigmoidal) nature of compressive flow curves (Figure 3) with higher work hardening observed for such specimens is typical of Mg metal matrix composites reinforced with solid particulate fillers [1]. These results observed in the present study are consistent with Ref’s [9,37], indicating a critical threshold of 30 vol % of GMB in the Mg matrix to exhibit syntactic foam behavior. Further, it can also be noted that Mg-5 and Mg-15 foams approach densification at strains of ~16 and ~18% respectively, indicating the complete collapse of the microballoons. With the addition of 25 wt % GMB, the densification strain was further increased to ~37%. This indicates the ability of GMB to resist the compressive force, and makes them suitable for many marine applications. Incorporating higher level of porosity in foams leads to a stable stress plateau in their plastic deformation regime, as observed in this study. The pores, which are surrounded by stiff, strong GMB walls, impede deformation and collapse during compressive loading, while further loadings of deformed material occupy the porosity opened up due to fracture. Loading furthers the progressive densification of the microstructure.

The compressive properties of the syntactic foams are shown in Table 2. The compressive yield strength (0.2% CYS) of Mg increased from 66 to 77, 102, and 161 MPa with the addition of 5, 15, and 25 wt % of GMB, respectively. The maximum ultimate compressive strength (UCS) was exhibited by Mg-5 of 232 MPa (~19.6% higher than pure Mg). The UCS of Mg-15 remained almost unchanged compared with Mg-5. However, by embedding an increased amount of porosity with the addition of 25 wt % GMB, UCS decreases to 216 Mpa, which was still ~11.3% higher than Mg. The compressive fracture strain of Mg/GMB foams increased with the progressive addition of GMB, with Mg-25 foam exhibiting the maximum fracture strain of 37.7%, which is significantly greater (~151%) than Mg. Apart from the mechanical properties of the metal matrix and the hollow particles, the compressive behaviour of MMSFs depend on volume fraction, structure, and the distribution of the microballoons [38]. The enhanced compressive properties of Mg/GMB syntactic foams obtained in this study can be attributed to the fact that the strength of the glass itself may be significantly higher than the Mg matrix. Several other reasons can also result in superior compressive properties of syntactic foams such as (a) the presence of fairly dispersed hard GMB and secondary phases; (b) effective transfer of load from the Mg matrix to GMB; (c) a mismatch coefficient of thermal expansion values leading to dislocation formation; and (d) Orowan strengthening due to the presence of secondary phase [16,39]. Moreover, the possibility of uniform pore size and content that can be precisely controlled by using engineered hollow particles such as GMB results in improved mechanical properties of the resulting foams as compared to MgMSFs containing fly ash cenospheres [37,39].

The complex concurrence of processes, extent of hollow particles breakage and plastic deformation of the matrix significantly influence the yielding behaviour of foams [40]. Load transfer mechanism has been identified as the major contributing factor affecting the strength in of MgMSFs [39]. At lower filler loadings, the yield strength of the Mg/GMB foams is dominated by the strength of the reinforcing phase, whereas with increased GMB content, both the glass spheres and Mg matrix contribute to the strength. Increase in the volume fraction of hollow GMB, and consequently an increase in the matrix–particle interface area leads to higher yield strength of the syntactic foams. This might be due to the particles contributing to stress accommodation, as observed from Table 2. Further, in situ formed Mg_2_Si phases present in the Mg matrix contribute to strengthening by increasing the dislocation density, effective stress transfer, and Orowan strengthening mechanism [41]. Similar observations have been made in the case of AZ91D/cenosphere syntactic foams, wherein the characteristics of the interface, such as interfacial reaction, resultant interfacial bond strength, and dislocations govern the compressive properties leading to CYS rise with the increasing Mg_2_Si content [42].

**Figure 4:**
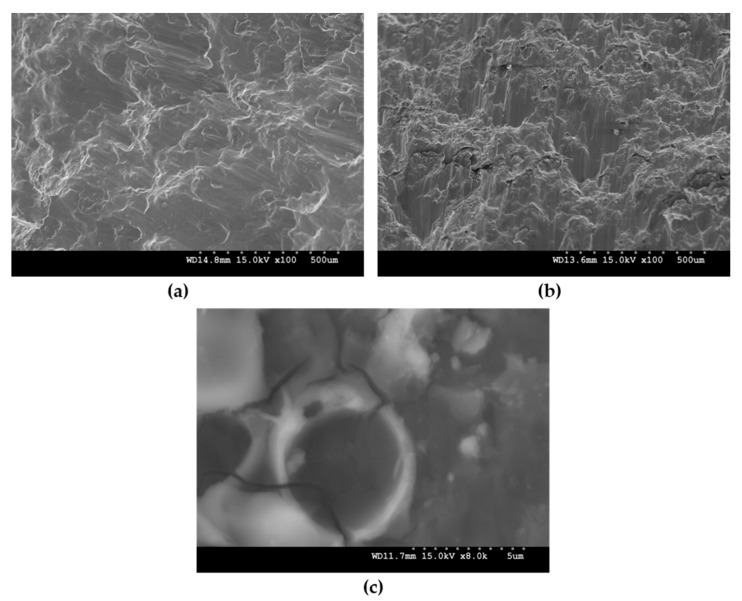
Representative fractographs after compressive loading of: (**a**) Pure Mg, (**b**) Mg-25 and (**c**) crack observed in a glass microballoon (GMB) particle during the plateau region.

The energy absorption (EA) during compressive loading increased with the GMB addition (Table 2). Mg-25 exhibited the maximum EA value of 63.4 MJ/m^3^, which is ~200% greater than that of pure Mg. Though the EA capabilities of Mg-5 and Mg-15 were ~34.6 and ~55% greater than Mg, considerable strain hardening effect is visible as the steep monotonic rise limits energy absorption efficiency, which facilitates the dissipation of high levels of energy. However, the addition of 25 wt % GMB resulted in a stress plateau region, indicating limited strain hardening during plastic deformation and resulting in enhanced energy absorption. The higher EA capabilities of Mg foams are attributed to plateau behaviour collapse wherein the sequential crushing of microballoons takes place and the material absorbs energy without any significant change in strength. The majority of EA is converted into plastic deformation energy [8]. During the plateau region, the crushing of GMB particles and failure of the matrix is clearly evident (Figure 4c), which initiate and cause particle failure. Structural densification results as GMB fail, leading to a soft compliant Mg matrix occupying the cavity space. Mg/GMB with such high energy absorption can outperform conventional foams due to higher specific strength, which makes them potential candidates for damping/energy absorbent components in automotive crash boxes replacing open cell foams. To confirm the mode of failure under compression, fracture studies were performed. Under compressive loading, fracture surfaces are at about 45 degrees with respect to the compression loading direction. Shear bands were clearly evident, as observed in Figure 4.

**Figure 5:**
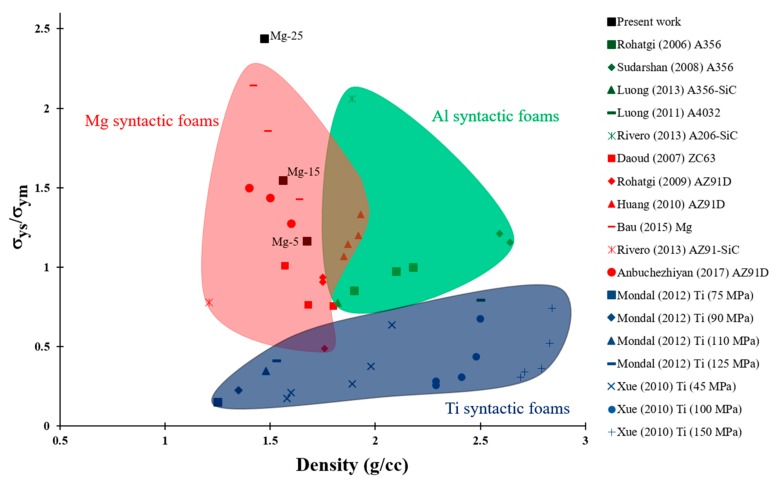
Normalized compressive yield strength of Mg/GMB syntactic foams with respect to composite density, compared with other metal matrix syntactic foams (MMSFs) from the published literature.

The weight-saving potential of syntactic foams can be realized by understanding the relationship between the syntactic foam density and mechanical properties. The compressive yield strength of ceramic reinforced syntactic foams normalized with the yield strength of the matrix material have been plotted with respect to composite density in Figure 5 to visualize the weight-saving potential. The data in the figure represent Al, Mg, and Ti matrix syntactic foams and are extracted from the literature [11,16,18,37,39,42,43,44,45,46,47,48]. It can be observed that the Mg/GMB syntactic foams synthesized in this study have the highest improvements in the yield strength with a significant reduction in density compared with other types of syntactic foams studied. It can also be noted that MgMSFs have higher values of yield strength and maintain their superiority over Al and Ti matrix syntactic foams. Al matrix syntactic foams have yield strength values from 30 to 70% lower than those of MgMSFs at a comparable density. Weight saving can be achieved in load-bearing applications by replacing the Al and Ti matrix syntactic foams with MgMSFs without compromising strength. The possibility of achieving a wide range of density values provides significant capabilities to material scientists and engineers based on the applications.

### 3.7. Elastic Modulus

Table 3 lists the elastic modulus measured for Mg and their foams. It can be observed that the syntactic foam exhibit lower modulus with increasing GMB content. Mg-25 exhibited the minimum value of 39.85 GPa (~8% lesser than that of pure Mg). In general, the addition of solid ceramic reinforcements may exhibit a higher modulus compared with Mg matrix, but at increased density. The uniform distribution of reinforcement, relative moduli difference of the constituents, and interfacial bonding between them prompts higher elastic moduli [1,37] in such composites. However, with the addition of thin-walled hollow particles such as GMB, as used in the present work, the modulus decreases as the air void inside the glass shell reduces the effective modulus of a hollow particle compared with that of a solid particle [49]. The effective modulus of thin-walled GMB used in the present work vary from 2.7–5.9 GPa, which is significantly lower than that of the glass material (60 GPa) [50]. As a result, with increasing GMB content from 5–25 wt %, the elastic modulus of pure Mg decreases from 43.3 to 39.85 GPa. Similar observations are noted in ZC63/cenosphere and AZ91D/cenosphere foams [37,39].

Even though Mg-based materials may not be suitable where high modulus is required, a range of applications are looked into in the biomaterials area in the recent past owing to its superior biocompatibility and biodegradability [51]. Mg exhibits elastic modulus (41–45 GPa) closer to that of human bone (3–30 GPa) in comparison with other biomaterials such as Ti (100–110 GPa) and stainless steel (189–205 GPa). Further, Mg doesn’t exhibit local or systemic toxicity [52]. Silica glass microspheres used in this study are also biocompatible, and can be used safely in applications requiring implantation [53]. Further, GMBs have already found applications in the controlled release delivery vehicle for antibodies, recombinant antibody derivatives, and small oligonucleotides, along with their use in the repair, restoration, and regeneration of tissue within the human body [53,54]. With the development of Mg/GMB syntactic foams, the elastic modulus of Mg can further be reduced, making them an viable option in orthopaedic applications such as implants and fixation devices. As a potential implant material, Mg/GMB syntactic foams can decrease the stress shielding effect by providing a close elastic modulus to the human bone and possibly eliminate further corrective procedures.

The compressive and elastic modulus properties can be collectively termed biomechanical properties. The biomechanical properties of developed Mg/GMB syntactic foams are compared with natural bone and cortical bone of human body along with Ti and steel in Table 3. From the results shown in Table 3 compiled from Ref [1,52,55], the 0.2% CYS, UCS, and elastic modulus for the Mg/GMB syntactic foams are closer to that of bone and bone tissues, and could improve the interface between these developed foams and bone cells when compared with currently preferred biomaterials such as Ti alloys and stainless steel. Depending on the requirements of an application, either syntactic foams of desired modulus or strength may be selected from this available composition range to widen the horizons of Mg in implant surgery.

## 4. Conclusions

Based on the present study, following conclusions are drawn:

1. Mg matrix syntactic foams with hollow glass microballoons (GMB) as a reinforcement can be successfully synthesized using disintegrated melt deposition technique. Mg-25 foam exhibited an average density of 1.47 g/cc similar to polymers signifying its weight saving potential and elevated temperature usage.
2. CTE values reduced with the incorporation of GMB in pure Mg with Mg-25 foam exhibiting the CTE value of 21.2 × 10^−6^/K indicating superior thermal and dimensional stability.
3. The ignition temperature of Mg-25 showed the highest resistance to ignition, with an increase in ignition temperature of ~22 °C. Further, the ignition temperature of the developed foams exhibit a perfect first-degree polynomial with respect to ignition factor, a new parameter proposed by authors.
4. The hardness of pure Mg increased with the increasing GMB content, with Mg-25 showing a maximum increase of ~127.7%.
5. Under compression loading, 0.2% CYS and compressive fracture strain of pure Mg increased with GMB addition. Mg-25 foam exhibited a 0.2% CYS and compressive fracture strain of ~161 MPa and ~37.7% respectively. However, the maximum UCS was observed in Mg-5 foam.
6. Energy absorbed under compressive loading also increased with progressive addition of GMB, with Mg-25 foam showing a significant improvement of ~200% as compared to pure Mg.
7. The superior compressive properties with elastic modulus closer to natural bone makes Mg/GMB syntactic foams a potential choice for implant materials.

## Figures and Tables

**Table 1:** Results of density, coefficient of thermal expansion (CTE) and the ignition measurement of Mg and Mg/GMB syntactic foam specimens.

Material	Theoretical Density (g/cc)	Measured Density (g/cc)	Matrix Porosity (vol %)	CTE (× 10^−6^/K)	Ignition Temperature (°C)
Mg	1.738	1.701 ± 0.002	2.1	27.1	590 ± 1.2
Mg-5	1.686	1.674 ± 0.015 (↓1.6%)	0.72	24.2 (↓10.7%)	602 ± 1
Mg-15	1.588	1.559 ± 0.010 (↓8.3%)	1.78	22.7 (↓16.2%)	609 ± 0.8
Mg-25	1.502	1.472 ± 0.018 (↓13.4%)	1.98	21.2 (↓21.7%)	612 ± 0.5

* (↓x%) indicates the decrease in the property with respect to pure Mg by x%.

**Table 2:** Results of hardness and room temperature compression testing.

Material	Hardness (Hv)	0.2% CYS (MPa)	UCS (MPa)	Ultimate Compressive Strain (%)	Energy Absorbed (MJ/m^3^)
Pure Mg	47 ± 2	66 ± 3.5	194 ± 8	15 ± 1	21.1 ± 1.2
Mg-5	82 ± 4 (↑74.5%)	77 ± 3 (↑16.7%)	232 ± 7 (↑19.6%)	17.2 ± 0.6 (↑14.7%)	28.4 ± 1.3 (↑34.6%)
Mg-15	91 ± 5 (↑93.6%)	102 ± 5 (↑54.5%)	231 ± 6 (↑19.1%)	19.1 ± 0.7 (↑27.3%)	32.7 ± 1.6 (↑55%)
Mg-25	107 ± 6 (↑127.7%)	161 ± 4 (↑144%)	216 ± 6 (↑11.3%)	37.7 ± 2 (↑151.3%)	63.4 ± 3.2 (↑200%)

* (↑x%) indicates the increase in the property with respect to pure Mg by x%.

**Table 3:** Elastic modulus measurements of Mg and their syntactic foams along with comparison of biomechanical properties.

Material	Density (g/cc)	0.2% CYS (MPa)	UCS (MPa)	Ultimate Compressive Strain (%)	Elastic Modulus (GPa)
Natural Bone	1.8–2.1	130–180	-	-	3–20
Cortical Bone	1.3 ± 0.03	-	131–224	2–12	15–30
Titanium alloy	4.4–4.5	1040	1643–2324	29–49	110–117
Stainless Steel	7.9–8.1	170–310	-	-	189–205
Pure Mg	1.7014	66	194	15	43.3
Mg-5	1.6739	77	232	17.2	42.56 (↓1.7%)
Mg-15	1.5597	102	231	19.1	41.10 (↓5.1%)
Mg-25	1.4723	161	216	37.7	39.85 (↓7.9%)
